# S15-2 Physical activity, sedentary behaviour, and cognitive function among older adults: a bibliometric analysis of literature from 2002 to 2022

**DOI:** 10.1093/eurpub/ckad133.073

**Published:** 2023-09-11

**Authors:** Zhen Yang, Pieter-Jan Marent, Pauline Hotterbeex, Jannique van Uffelen

**Affiliations:** KU Leuven, Belgium; KU Leuven, Belgium; Ghent University, Belgium; KU Leuven, Belgium; Ghent University, Belgium; KU Leuven, Belgium

## Abstract

**Purpose:**

Physical activity and sedentary behaviour have received intense interest in recent decades from a variety of stakeholders due to their diverse association with cognitive function, which is a key component in maintaining health of older adults. The aim of this bibliometric analysis is to reveal the developmental structure, research hotspots, and predict future trends in the field of physical activity, sedentary behaviour, and cognitive function research among older adults.

**Methods:**

This study adhered strictly to the step-by-step guideline of bibliometric analysis. Publications in this field from 2002 to 2022 were retrieved from the Web of Science Core Collection. Only original articles and review articles published in English were included in the analysis. A cubic polynomial was applied to determine the publication trends. CiteSpace (6.1.R4 Basic) and VOSviewer (1.6.18) were performed to obtain collaborative networks, reference co-cited networks, and keyword co-occurrence networks, as well as to perform burst analysis to predict future trends.

**Results:**

A total of 1093 publications were retrieved, of which 73.56% obtained research grants. The publications were cited 30,317 times. Within two decades there was rapid growth in publications in this field. The USA (329 items, 30.10%) and China (136 items, 12.44%) were the top contributors, and the USA had the strongest collaborative network. University of British Columbia (41 items, 3.75%) and University of California System (40 items, 3.66%) were the most active institutions. Liu-Ambrose T (32 items, 2.93%) and Shimada H (20 items, 1.83%) contributed to the largest number of publications. Journal of Aging and Physical Activity, and Frontiers in Aging Neuroscience were the most active journals. Reference co-cited networks, and keyword co-occurrence networks revealed that hotspots in this field were age, nursing home resident, tai chi, and perceptions, while future trends in this field were healthy ageing, qi gong, sedentary behaviour, and falls.

**Conclusions:**

There has been an increased research interest in the interrelationships between physical activity, sedentary behaviour, and cognitive function in older adults in the past two decades. Our findings will help researchers, practitioners, and other stakeholders to understand the structure and hotspots in this relevant field of research.

**Funding:**

Not applicable.

